# The Relationship between *Pneumocystis* Infection in Animal and Human Hosts, and Climatological and Environmental Air Pollution Factors: A Systematic Review

**DOI:** 10.21926/obm.genet.1804045

**Published:** 2018-10-26

**Authors:** Robert F. Miller, Laurence Huang, Peter D. Walzer

**Affiliations:** 1. Centre for Clinical Research in Infection and Sexual Health, Institute for Global Health, University College London, London WC1E 6JB, UK; 2. Clinical Research Department, Faculty of Infectious and Tropical Diseases, London School of Hygiene and Tropical Medicine, London WC1E 7HT, UK; 3. Bloomsbury Clinic, Mortimer Market Centre, Central & North West London NHS Foundation Trust, London WC1E 6JB, UK; 4. HIV Services, Royal Free London NHS Foundation Trust, London NW3 2QG, UK; 5. Division of Pulmonary and Critical Care Medicine, Zuckerberg San Francisco General Hospital and Trauma Center, University of California, San Francisco, CA 94110, USA; laurence.huang@ucsf.edu; 6. HIV, Infectious Diseases, and Global Medicine Division, Zuckerberg San Francisco General Hospital and Trauma Center, University of California, San Francisco, San Francisco, CA 94110, USA; 7. Department of Internal Medicine, University of Cincinnati, Cincinnati, OH 45267, USA; peter.walzer1@gmail.com

**Keywords:** Pneumocystis, Season, Temperature, Climate, Environment, air pollution, detection

## Abstract

**Background:**

Over the past decade, there has been rising interest in the interaction of *Pneumocystis* with the environment. This interest has arisen in part from the demonstration that environmental factors have important effects on the viability and transmission of microbes, including *Pneumocystis*. Environmental factors include climatological factors such as temperature, humidity, and precipitation, and air pollution factors including carbon monoxide, nitrogen dioxide, sulfur dioxide, and particulate matter.

**Methods:**

We undertook a systematic review in order to identify environmental factors associated with *Pneumocystis* infection or PCP, and their effects on human and animal hosts.

**Results:**

The systematic review found evidence of associations between *Pneumocystis* infection in animal and human hosts, and climatological and air pollution factors. Data from human studies infers that rather than a seasonal association, presentation with PCP appears to be highest when the average temperature is between 10 and 20°C. There was evidence of an association with hospitalization with PCP and ambient air pollution factors, as well as evidence of an effect of air pollution on both systemic and bronchoscopic lavage fluid humoral responses to *Pneumocystis*. Interpretation of human studies was confounded by possible genetically-determined predisposition to, or protection from infection.

**Conclusions:**

This systematic review provides evidence of associations between *Pneumocystis* infection in both animal and human hosts, and climatological and environmental air pollution factors. This information may lead to an improved understanding of the conditions involved in transmission of *Pneumocystis* in both animal and human hosts. Such knowledge is critical to efforts aimed at prevention.

## 1. Introduction

*Pneumocystis jirovecii* is a fungus that continues to be an important cause of pneumonia (PCP) in the immunocompromised host and a major cause of death in humans [1]. It is estimated that there are more than 400,000 annual cases of PCP worldwide, with over 52,000 deaths per year [2]. Knowledge of the basic biology of *Pneumocystis* has long been limited by of the lack of a reliable and reproducible method of in vitro cultivation [3] but important insights have been gained from both animal and human studies. *Pneumocystis* organisms found in different hosts are morphologically indistinguishable but host species-specific [1]. Colonization of apparently healthy asymptomatic humans may provide a reservoir of *P. jirovecii*, and transmission of *Pneumocystis* to both susceptible and healthy persons may occur. However, the exact relationship is incompletely understood, and environmental reservoirs for the organism have also been suggested. *Pneumocystis* infection is acquired by inhalation, and the infective moiety is the cystic form [4] but the precise conditions for airborne transmission are unknown. Traditionally, PCP was thought to be the result of reactivation of latent infection acquired early in childhood, but molecular studies from PCP outbreaks demonstrate that PCP can also result from recent exposure in an at-risk host [5]. As a result, an improved understanding of the conditions involved in transmission is critical to efforts at prevention.

Over the past decade, there has been rising interest in the interaction of *Pneumocystis* with the environment. This interest has arisen in part from the demonstration that environmental factors have important effects on the viability and transmission of microbes, including *Pneumocystis*. Studies have demonstrated an association between environmental factors and the risk of PCP as well as specific antibody responses against *P. jirovecii*. The environmental factors can broadly be divided into two groups: a) climatological factors such as temperature, humidity, and precipitation; and b) air pollution factors including carbon monoxide (CO), nitrogen dioxide (NO_2_), sulfur dioxide (SO_2_) and particulate matter [6]. We undertook this systematic review in order to identify specific environmental factors associated with *Pneumocystis* infection or PCP, and their effects on human and animal hosts. Our goal was to comprehensively review the published literature on this topic in order to gain insights and advance our understanding of this important human pathogen.

## 2. Materials and Methods

We sought to identify publications describing associations between environmental factors and detection of *Pneumocystis* in humans and animals, and with development and presentation with PCP. We first reviewed English-language published articles of *Pneumocystis* and PCP and associated climatological and air pollution factors for the period 01 January 1960 to the present day (30 June 2018) using PubMed (US National Library of Medicine). The following search terms were used: Pneumocystis [Title] + English [Language], then Human [MeSH], then one of the following MeSH: season, climate, air pollution, environment, geography, humidity, or temperature. In addition, we reviewed the references within each publication for additional articles. Since the first reports of human immunodeficiency virus (HIV)-associated PCP in the early 1980s, most cases of PCP in the literature have been described in the context of HIV infection.

The scope of the present study was increased to include animals studied in the wild, in slaughterhouses, and in research laboratories. The same time period was used and the following search terms were used in the literature search: Pneumocystis [Title] + English [Language], then, Animal [MeSH], then one of the following MeSH: season, climate, air pollution, environment, geography, humidity, or temperature. Inclusion of these animal studies was done in order to enhance the findings from human studies and to provide important insights that add to our understanding of studies in humans.

Studies identified by the search strategy were divided into three groups:

Group 1: animal studies. Group 2: human studies of HIV-infected and uninfected patients with PCP divided based on geographic location. Group 3: human studies of HIV-infected patients with PCP associated with ambient air pollutants.

Where specific climatological information (i.e., temperature and humidity/precipitation) was not described in a specific publication we used World Weather Online [7]. In addition, as this is a systematic review of previously published work, Ethics Committee/IRB approval was not required from any of the three centers involved in this work.

## 3. Results

### 3.1. Literature Search

**Figure 1:**
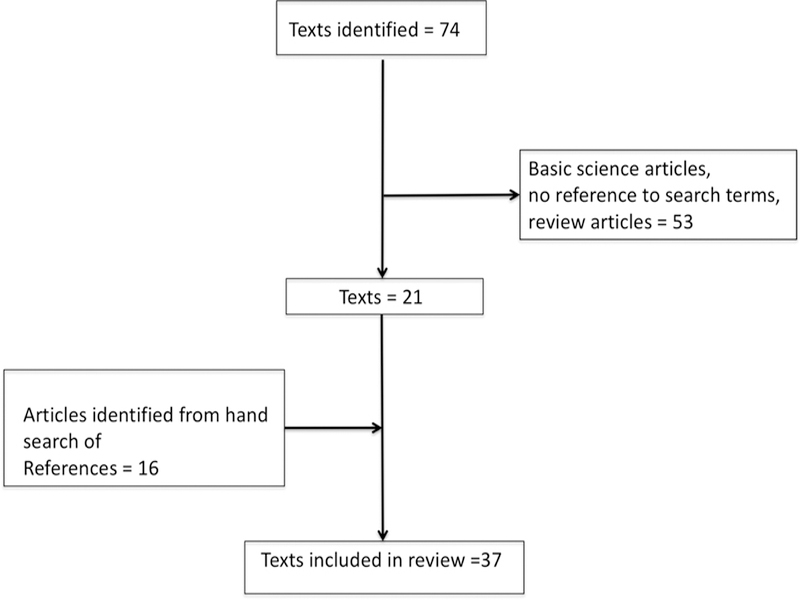
Results of literature search.

The PubMed literature search for the period 01 January 1960 through 30 June 2018 identified 74 unique full text articles. From these, 53 articles were excluded (after review of the full text by LH and RFM), as they were not relevant to the topic, or they were review articles that did not contain original data. In addition to the 21 remaining articles, 16 articles were identified by hand searching by the authors (LH and RFM). Additionally, the authors of some publications identified either by the MeSH search or via the “hand search” were contacted (by RFM) in order to obtain additional information not contained in their original published manuscript. Thus, in total 37 articles were identified and were included in the systematic review. Figure 1 shows the results of the literature search.

### 3.2. Animal Studies Showing Associations between Detection of Pneumocystis and Seasonal and Environmental Factors

Group 1. Eleven studies in animals (six from Europe, two from South America, two from Asia, and one from USA) were identified. Animal hosts were pigs (four studies), wild mouse species, shrew species, field voles, rats, hares, crab-eating macaques, and bats (each one study), (Table 1) [8–18]. *Pneumocystis* was detected in all of the animal species studied, using a range of techniques including Grocott silver staining, immunohistochemistry, and polymerase chain reaction (PCR). Using PCR, *Pneumocystis* was detected in as few as 4–5% of pigs in a slaughterhouse [17] to as many as 34.5% of crab-eating macaques living in a Primatology Center [12].

The main findings were that in eight of the 11 studies there was a clear seasonal variation in detection of *Pneumocystis* among different hosts, in their natural surroundings [8, 9, 10, 11, 14, 15, 17, 18]. In the study of macaques housed in a Primatology Center, detection was associated with mean precipitation, and in the study of rats in a laboratory facility, temperature and humidity had a clear influence on the predominance of different types of *Pneumocystis*. Finally, the study of bats in both captive and wild environments showed no association between detection of *Pneumocystis* and either temperature or humidity, but an association with smaller, crowded sedentary colonies at altitudes below 800m above sea level.

### 3.3. Human Studies Showing Associations between Pneumocystis and Seasonal and Environmental Factors

Group 2. Twenty-four studies in HIV-infected and uninfected patients with PCP were identified and were divided, based on geographic location. Seven studies were from USA, two from South America, 11 from Northern Europe, three from Southern Europe, and one from Australia, (Table 2) [6, 19–41]. These studies were a mixture of prospective and retrospective clinical or laboratory (including autopsy) studies and were national, multi-center or single center in their design. Two studies were done in infants, the remaining 22 studies were done in adults. Among the adult studies 19 included only HIV-infected adults, two included HIV-infected and uninfected adults, and one included HIV-uninfected adults with underlying rheumatologic conditions. In the two studies of infants, subjects were HIV-uninfected [19–41].

The main findings were that in 18 studies in adults there was evidence of a seasonal and/or climatological association and presentation with PCP, or detection at autopsy and two studies showed no such association but noted clustering of cases of PCP by Zip Code, and another identified that time spent outdoors was associated with risk of PCP. In the remaining two studies there was no apparent seasonal or climatologic association with development of PCP. However, both studies showed an apparent ethnic predisposition, with patients of black African origin being at reduced risk of developing PCP compared to patients who were of Western origin (Table 2). Both studies in infants showed a seasonal variation either in detection of *P. jirovecii* at autopsy, or variation in antibody responses to *Pneumocystis* major surface antigen components.

### 3.4. Human Studies Showing Associations between Pneumocystis and Ambient Air Pollution Factors

Group 3. Four studies (three from USA, one from Spain) reported the impact of ambient air pollution factors among HIV-infected adult patients hospitalized with PCP (Table 3) [6, 40, 42, 43]. Of note two of these four studies are also included in Group 2 [6, 42]. The San Francisco studies were single-center, prospective studies while the study from Spain was a national study.

The main findings were that one study showed elevated levels of NO_2_, PM_10_ and ozone in ambient air were associated with increased risk of hospitalization with PCP [40], and in another SO_2_, was associated with increased risk of hospitalization, but the risk was attenuated by elevated CO levels [6]. Two further studies that examined serologic responses in hospitalized patients with PCP, and showed elevated NO_2_, and PM_10_ were independently associated with impaired IgM responses to *P. jirovecii* major surface glycoprotein (Msg) constructs in serum [42], and that there was an impaired IgA response to *P. jirovecii* Msg in bronchoscopic lavage (BAL) fluid that was associated with increased ambient ozone exposure. Additionally, increased BAL fluid IgA responses were associated with increased ambient NO_2_ exposure [43] (Table 3).

## 4. Discussion

We believe that the present study is the first systematic review of the relationship between *Pneumocystis* infection in its animal and human hosts, and the effects of climatological and air pollution factors in the environment on this relationship. This review found evidence of associations between *Pneumocystis* infection in animal and human hosts, and climatological and environmental air pollution factors, but the quality of evidence was poor and was limited by inconsistent and incomplete sampling methodology in both animal and human studies.

### 4.1. Non-Pneumocystis Fungi – Seasonal and Climatological Factors

Climatological factors, including temperature and humidity, are associated with variations in concentrations of ascomycetes, basidiomycetes, and other fungi in air [44–48]. A study from Porto Alegre, Southern Brazil, showed the highest detection of airborne fungal spores was in summer (December-February), when average minimum air temperatures are typically between 18 and 21°C [7]. The lowest rates of detection were in the autumn (March-May), when average minimum temperatures are typically between 12 and 17°C [45]. A second study from Fortaleza, North East Brazil, where the climate is hotter, reported seasonal variation in rates of detection of airborne fungi, (higher rates of detection were observed in January-April, and lower rates were recorded in July-October [46]. This city experiences a rainy season (February-July: rainfall 1883.5 mm, average temperature 26.8°C) and a dry season (August-January: rainfall 260 mm, average temperature 27.6°C) [46].

### 4.2. Pneumocystis - Seasonal Factors – Average Temperature

In the studies of *Pneumocystis* in animals studied in the wild, in slaughterhouses, and in research laboratories that were identified in this systematic review interpretation of the observed seasonal variation is confounded by the fact that none of the studies systematically sampled throughout the year, nor sampled in consecutive years, and none of the studies included “controls”. Thus, possible alternative explanations for the apparent seasonal variation must include closer animal-to-animal proximity within animal colonies, driven by climatological factors, and by season, for example by closer co-habitation during the mating season for an individual animal host. This potentially increases the chance for airborne or “other” animal-to-animal transmission, and thus likelihood of detection by opportunistic, or systematic sampling. In the studies of pigs, another factor influencing detection of *Pneumocystis* is the type of husbandry used in rearing the animals, as higher rates of detection were observed in semi-intensively farmed animals when compared with pigs who were intensively-farmed. This finding has been ascribed to less control of production variables in pigs reared semi-intensively [17]. Additionally, detection of *Pneumocystis* has been associated with underlying viral and/or bacterial infection in pigs. It has been suggested that these infections may act as potential “immune suppressants” thus permitting colonization/infection with *Pneumocystis* [15, 17]

Taken together the human studies clearly demonstrate evidence of a seasonal and/or climatological association and presentation with PCP, or serologic or autopsy detection of infection. A seasonal variation in incidence of PCP was first reported in 1991 [19]. Subsequently, the relationship between seasonality and temperature and hospitalization with PCP has described inconsistent findings with some, but not all studies describing any association. In both HIV-infected and uninfected patients the risk of PCP has been observed to be higher in summer (London, UK) [28, 35], (Geneva, Switzerland [29] (Munich, Germany) [36] and (San Francisco, USA) [6] autumn (Melbourne, Australia) [41], and winter (Seville, Spain) [40]. As previously suggested [6], these differing results might be as a result of differences in patient populations, climatological factors, geography, *Pneumocystis* genotypes, or to differences in study design [6]. Because of geographical climatological differences, it is likely that summer temperatures in one country, or region, are similar to autumn or winter temperatures in another geographical location. For example, in Spain winters are generally mild (most regions having an average temperature between 10 and 20°C), and summers are hot (with average temperatures greater than 30°C) [40]. Thus, the average temperature in a Spanish winter is similar to that observed in other seasons in other countries during which the highest incidence of PCP has been described [40]. In London the “peak” summer temperature was 13°C *28], the mean summer temperature in San Francisco was 17.6°C [6], and average temperature in Melbourne was between 13.2 and 20°C [7, 40]. Taken together, interpretation of these data, infer that rather than a seasonal association, presentation with PCP appears to be highest during the season of the year when the average temperature is between 10 and 20°C [40]. As previously noted [40], among HIV-infected persons, other confounding factors, including tobacco smoking [24], chronic obstructive pulmonary disease, bacterial pneumonia, and colonization of the respiratory tract by *P. jirovecii*, can contribute to increased risk of developing PCP, and also that these contributing factors additionally, are potentially affected by climatological factors [24].

A similar seasonal association is apparent in the two studies of infants done in Chile [25, 26]. In the autopsy study the highest rate of detection of *Pneumocystis* was in winter (June-August), when average maximum temperatures were 16–18°C, and the lowest rate of detection was in autumn (March-May), when average maximum temperatures were 20–28°C [7, 25]. In the study of serum antibody responses to *Pneumocystis* MsgA constructs among immune competent infants the lowest peaks were also detected in autumn (March-May) [26].

### 4.3. Seasonal Factors – Humidity and Precipitation

There is a less certain association between the climatological variables of humidity and precipitation, and detection of *Pneumocystis* in animal and human hosts, or with presentation with PCP in humans. Some animal studies [12, 17, 18] and some human studies [20, 27, 28, 33] suggest an association, but others do not. Confounding these observations is the fact that data about this climatological variable was not routinely collected in either the animal or human studies.

A further potential factor, confounding interpretation of data concerning both temperature and humidity/precipitation in humans is that in the EuroSIDA study, which was a prospective observational cohort study that reported diagnosis of PCP in North, Central, and South Europe, the higher prevalence of PCP described in cooler, wetter North European climates might be explained by other logistical factors, including better access to healthcare (and so the likelihood of being diagnosed with PCP) in North Europe [30]. A similar interpretation can be applied to the findings from another European retrospective multicenter study [31].

### 4.4. Seasonal Factors – Outdoor Activities

The study from Atlanta, USA showed spending time outdoors gardening, camping or hiking in the six months prior to hospitalization with pneumonia was strongly associated with risk of PCP [21]. Additionally, studies from Cincinnati and San Francisco, USA showed clustering of PCP by Zip Codes [22, 24]: the former study showing more cases in affluent areas with more green space [22], the latter reporting more cases in residential areas containing parks and small yards [24]. Taken together, these three studies infer that susceptible individuals are at greater risk of developing PCP if they have increased opportunities for being outdoors, and thus possibly for increased environmental exposure to *Pneumocystis*.

### 4.5. Genetic Factors

Observations from national (Netherlands) and single-site London (UK) cohorts infer that there might be an ethnic/genetically-determined predisposition to development of PCP [32, 37]. Single point mutations (SNPs) in genes associated with innate immune function are increasingly recognized as significant factors dictating an individual’s susceptibility to infection *^49^-^51^]. Two reports describe an association between SNPs and development of PCP in HIV-infected individuals [52,53]. The Fc*γ*IIa receptor, which binds to immune complexes to facilitate uptake of microbes, is encoded by a gene with synonymous (functional) polymorphisms that affects binding affinity to IgG. Participants in the Multicenter AIDS Cohort Study (MACS) with the FcγRIIa RR genotype progressed to AIDS (i.e., CD4 count <200 cells/uL) faster than participants with RH or HH genotypes [52]. By contrast, participants with an Fc*γ*RIIa HH genotype progressed more quickly to AIDS (defined by development of PCP) than those with other genotypes [52]. While the underpinning mechanisms remain uncertain, these results raise intriguing questions about the antibodies that opsonize *Pneumocystis* and interact with Fc*γ*RIIa receptors. The chemokine receptor CXCR6 is a co-receptor that facilitates fusion of HIV to CD4 cells. The ligand for CXCR6 (CCXCL16) is highly expressed in the lungs. A SNP in codon 3 (CXCR6-E3K) is common in African Americans and rare in Caucasians. The AIDS Link to Intravenous Experience (ALIVE) study, a prospective cohort study of predominantly African American HIV-infected drug users, examined the relationship of CXCR6 SNPs and development of PCP [53]. Time to development of PCP was similar among the genotypes, but subjects homozygous or heterozygous for CXCR6–3E were more likely to die after PCP (and thus had a shorter survival time) than subjects homozygous for CXCR6–3K [53].

The current epidemiology of pediatric PCP in the USA demonstrates that it is less frequently observed in HIV-infected children. By contrast cases associated with hematologic malignancy and primary immunodeficiency have become more prominent, infants being the most commonly affected [54]. Well-described mutations in MHC class II (bare lymphocyte syndrome), recombination activating genes (RAG) −1 and −2, signal transducer and activator of transcription (STAT) −3, and IL-21R that can be modeled in genetically engineered mice and *P. murina* infection, provide evidence of genetic susceptibility to *Pneumocystis* infection [55]. Taken together these data suggest a gene-environment interaction in the disease.

### 4.6. Air Pollution

Among the general population it is increasingly evident that ambient air pollution contributes to the global burden of respiratory disease, including asthma, Chronic Obstructive Pulmonary Disease, and pneumonia [56–65]. The studies identified in this systematic review originating from Spain and San Francisco, USA clearly demonstrate an association between ambient air pollution factors and presentation of HIV-infected adults with PCP [6, 40]. It is intriguing that in one study there was no association with PM_10_, NO_2_, CO, or ozone, and an association was only evident for SO_2_ [6]. In the other study NO_2_, PM_10_, CO, and ozone were associated with risk of hospitalization with PCP [40]. This apparent difference may in part be explained by the fact that San Francisco is one of the least polluted cities in the United States and so it is possible that levels of these pollutants were below thresholds that can trigger respiratory complications [6].

The mechanisms by which ambient air pollution increases susceptibility to pulmonary infection are not well defined. Controlled exposure studies of single pollutants that have used cells, animals and human subjects indicate that ambient air pollutants alter innate lung immunity at multiple levels, including altered muco-ciliary function, respiratory epithelial cell dysfunction, impaired alveolar macrophage phagocytosis, and dysfunction of surfactant protein A and D [Reviewed in 6]. However, the effects of ambient air pollution factors on humoral immunity and serologic responses to pulmonary infection, and the immune-toxic effects of real-life exposures to ambient air pollution factors are poorly understood. In one study from San Francisco [42] PM_10_ and NO_2_ were independently associated with suppressed IgM (but not IgG) responses to *Pneumocystis* Msg constructs. It was suggested that PM_10_ particles encountering bronchus associated lymphoid tissue, might impair antigen presenting cell function, resulting in decreased activation of the humoral immune system and suppressed serologic responses [42]. Both animal and human exposure studies have found mixed effects of NO_2_ inhalation on bronchoalveolar and systemic antibody responses [66–69]. In a second study also from San Francisco patients with PCP increasing exposure to ozone was associated with reduced BAL fluid IgA responses to *P. jirovecii* Msg constructs and increasing exposure to NO_2_ was independently associated with increased BAL fluid IgA responses to *P. jirovecii* Msg [43]. These results might be explained by the observation that ozone is a potent oxidant resulting in both bronchoalveolar, and systemic inflammation. Animal studies have found that rats in the first two weeks of exposure to ozone demonstrate decreased antibody responses to microbial antigens such as *Listeria* spp [70].

## 5. Conclusions

This systematic review found evidence of associations between *Pneumocystis* infection in both animal and human hosts, and climatological and environmental air pollution factors. These data are limited by inconsistent and incomplete sampling methodology in both animal and human studies. Data from human studies infer that rather than a seasonal association, presentation with PCP appears to be highest when the average temperature is between 10 and 20°C. A potential confounder is possible genetically-determined predisposition to, or protection from infection. There is evidence of an association with hospitalization with PCP and ambient air pollution factors, as well as a clear effect of air pollution on both systemic and bronchoscopic lavage fluid humoral responses to *Pneumocystis*. The results of this systematic review provide an improved understanding of the conditions involved in transmission of *Pneumocystis* in both animal and human hosts. Such knowledge is critical to efforts aimed at prevention of infection.

## Figures and Tables

**Table 1:** Animal studies showing associations between detection of *Pneumocystis spp* and seasonal and environmental factors.

Study authors	Year	Species	Location	Methods	Main findings
Šebek & Rosický [8]	1967	Shrew species (*Sorex araneus*, *Sorex alpinus*, and *Neomys fodiens)*	Multiple rural locations, Czechoslovakia.	Grocott silver staining	*Pneumocystis* identified in 7/44 (16%) of shrews, only in Spring, and not in Autumn.
Poelma & Broekhuizen [9]	1972	Hare (*Lepus europaeus*)	Research Institute of Nature Management, Arnhem, Netherlands.	Grocott silver staining	*Pneumocystis* identified in 75/437 (17%) of hares, most commonly identified during the period September to December.
Shiota, Kurimoto & Yoshida [10]	1986	Wild mouse species (*Apodemus speciosus, Apodemus argenteus, Microtus montebelli, Mus musculus*)	6 localities, Japan.	Grocott silver staining	*Pneumocystis* detected in 11/142 (7.7%) of *Apodemus speciosus*; higher detection rate in Winter-Spring than in Summer-Autumn.
Laakkonen, *et al* [11]	1999	Field vole *(Microtus agrestis*) & Common shrew (*Sorex araneus*)	Rural Central (Luhanka) and Southern (Evo) Finland.	Grocott silver staining	Highest rates of *Pneumocystis* detection in both *Microtus agrestis* and *Sorex araneus* seen in November.
Demanche, *et al* [12]	2003	Crab-eating macaque *(Macaca fascicularis*)	Primatology Center, Strasburg, France.	PCR	Detection of *Pneumocystis* in 166/481 (34.5%) macaques: higher detection rate associated with mean precipitation rates.
Icenhour, *et al* [13]	2006	Brown Norway & Long Evans rats	Laboratory facility, Cincinnati, USA.	PCR	Higher relative humidity associated with predominance of *P. carinii,* higher temperatures associated with mixed infections of *P. carinii and P. wakefieldiae* and lower temperature associated with predominance of *P. wakefieldiae.*
Sanches, *et al* [14]	2007	Pigs	Slaughterhouses, in Rio Grande do Sul, and Mato Grosso, Brazil.	Grocott silver staining Immunohi sto- chemistry	*Pneumocystis* detected in 208/546 (36.9%) of pigs slaughtered in February-March (summer). Detection rate higher (39.9%) in slaughterhouse with a lower average temperature (Rio Grande do Sul: 23**°**C) than in Mato Grosso slaughterhouse (27**°**C).
Kim, *et al* [15]	2011	Pigs	Farms on Jeju Island, Korea.	Immunohi sto-chemistry	*Pneumocystis* detected in 39/172 (22.7%) of pigs. More commonly detected in cold season (December-March: temperature 8–9**°**C), than in hot season (June-September: temperature 21–23**°**C). Detection of *Pneumocystis* associated with PCV2 and PRRSV infection; in 32/139 (23%) virus positive, and in 4/28 (14%) of virus negative pigs.
Akbar, *et al* [16]	2012	Bats (New World and Old World microchiropters and Old World megachiropters)	New World (Mexico, Guyana, and Argentina wild bats and Old World (France) wild and captive bat colonies.	PCR	*Pneumocystis* identified in lungs of 71 of 216 (32.9%) of 19 bat species. More commonly detected in smaller, sedentary and crowded bat colonies. Also in colonies at <800m elevation. No association with geographical (cave) temperature or relative humidity.
Esgalhado, *et al* [17]	2013	Pigs	Slaughterhouses in the Lisbon and Tagus valley, Portugal.	PCR	*Pneumocystis* detected in 14/215 (7%) of pigs. Detection rates higher in pigs raised in Center and Algarve regions (10–13%), than in pigs from Lisbon and Alentejo regions (4–5%). No association with median temperature; association with extremely low and high precipitation rates. Detection more likely in semi-intensively farmed pigs (10%), than in intensively farmed pigs (5%)
Weissenbache r-Lang, *et al* [18]	2016	Pigs	Institute of Pathology and Forensic Veterinary Medicine, Vienna, Austria.	ISH/PCR	110 *Pneumocystis* positive pigs with pneumonia; many also had viral (PCV2, PRRSV, TTSuV1, TTSuV2), and/or bacterial (*B. bronchiseptica*, *P. multocida*, *M. hyopneumoniae*) infection. 79% of moderate and severe *Pneumocystis* seen in Winter-Spring (December-May).

Key: PCR = polymerase chain reaction; ISH = in situ hybridization; PCV2 = porcine circovirus type 2; PRRSV = porcine reproductive and respiratory syndrome virus; TTSuV1, TTSuV2 = torque teno sus virus type 1 and 2.

**Table 2:** Human studies showing associations between *Pneumocystis* and seasonal and environmental factors.

Region/ Study authors	Year	Location	Methods	Main findings
*USA*				
Hoover, *et al* [19]	1991	MACS cohort: Four US cities	Four centre retrospective cohort study.	Incidence of PCP greater in colder cities (Chicago & Pittsburgh) than in warmer cities (Baltimore & Los Angeles): PCP diagnoses peaked in May - June and were lowest in November - December.
Bacchetti [20]	1994	USA	Analysis of monthly trends in AIDS diagnoses among adolescents and adults reported to CDC.	Seasonal pattern of PCP, with a peak in March. More PCP in MSM/bisexual men in summer in West of USA (includes San Francisco and Los Angeles) which is drier than rest of USA at this time.
Navin, *et al* [21]	2000	Atlanta, Georgia	Two centre single city retrospective cohort study.	PCP associated with outdoor activities (gardening, hiking or camping).
Dohn, *et al*[22]	2000	Cincinnati, Ohio	Single centre cohort study.	Clustering of PCP cases by Zip code. Most cases occurred in affluent areas with more green space. No seasonal variation.
Morris, *et al* [23]	2000	San Francisco, California	Single centre cohort study.	Clustering of PCP cases by Zip code. Most cases occurred in more residential areas that contain parks and small yards. No seasonal variation.
Morris, *et al* [24]	2004	MACS cohort Four US cities	Retrospective cohort study of patients who had necropsy. PCR detection of P. *jirovecii*.	Rates of colonization (determined by PCR) greater in cigarette smokers. Detection rates higher in Chicago and Pittsburgh (70.4% and 61.5%) than in Baltimore and Los Angeles (42.3% and 16%).
Djawe, *et al* [6]	2013	San Francisco, California	Single centre cohort study.	Seasonal variation in hospitalization with PCP; peak in summer (June-August).
*South America*				
Vargas, *et al* [25]	2005	Santiago, Chile	Autopsy study, two children’s hospitals. PCR detection of *P. jirovecii*.	*P. jirovecii* DNA detected in 51.7% of immune competent infants dying in the community and in 20% dying in hospital. Seasonal variation in detection: 53% in Winter (June - August) when maximum temperature = 16–18°C. and 30% in Autumn (March - May) when maximum temperature =20–28°C.
Djawe, *et al* [26]	2010	Santiago, Chile	Prospective cohort study.	Seasonal variation in serum antibody titers to *P. jirovecii* among immune competent infants. MsgA antibody titers highest in Spring (September - November) and lowest in Autumn (March- May) MsgC titers highest in Summer (December - February) and Winter (June - August)
*Northern Europe*				
Setnes & Genner [27]	1986	Copenhagen, Denmark	Two hospital autopsy study.	*Pneumocystis* identified in 83/1762 (4.7%) of autopsies at Righospitalet and Finseninstituttet Hospitals 1979–1984. Lower detection of *Pneumocystis* observed in 1981 corresponded with low temperature, low vapor pressure, and low humidity. Higher rate of detection in 1982–3 corresponded with higher average temperature and vapor pressure.
Miller, Grant & Foley [28]	1992	London, UK	Single centre retrospective cohort study.	Seasonal variation in PCP: highest in June-July and September, following periods with low rainfall and temperatures <13**°**C.
Vanhems, Hirschel & Morabia [29]	1992	Geneva, Switzerland	Single centre retrospective cohort study.	Seasonal variation in PCP cases: highest in June - September. Seasonal variation no longer observed after introduction of PCP prophylaxis.
Lundgren, *et al* [30]	1995	Copenhagen, Denmark	Multicenter prospective study in Northern, Central and Southern Europe.	Incidence of PCP higher in cooler climates (Northern Europe), compared with Central or Southern Europe.
Delmas, *et al* [31]	1995	Paris, France	Multicenter retrospective study in eight European countries and in Amsterdam, Netherlands.	Incidence of AIDS-defining PCP higher in Germany, Switzerland, and UK (Northern/Central Europe), and lowest in Portugal and Italy (Southern Europe).
Del Amo, *et al* [32]	1998	London, UK	Eleven center retrospective cohort study.	PCP was presenting AIDS-defining condition in 52/313 (17%) of black Africans, compared with 52/314 (34%) of non-Africans living in London.
Lubis, *et al* [33]	2003	London, UK	Single centre retrospective cohort study.	Variation in monthly incidence of PCP: highest in January (16.9%) associated with low monthly rainfall (<35 mm/month). January peak more prominent in some years and not evident in other years. Other peaks in April (9.8%) and September (9.6%) not associated with rainfall.
Miller, *et al* [34]	2007	London, UK	Single centre retrospective cohort study. Genotyping of *P. jirovecii* isolates at mt LSU rRNA locus.	Association between Genotype 2 and mixed genotypes, and season/month: peaks in June - July, and May - June and September, respectively. No association between temperature, or rainfall and specific genotypes.
Walzer, *et al* [35]	2008	London, UK	Single centre retrospective cohort study.	Seasonal variation in presentation of PCP over a 21 year period: highest in summer (June - August; 29.8%) and lowest in winter (December - February; 21.9%).
Sing, *et al* [36]	2009	Munich, Germany	Single centre cohort study.	Seasonal variation in cases of PCP: peak in summer (May - August). Mean temperature (but not rainfall or wind strength) associated with incidence of PCP.
Schoffelen, *et al* [37]	2013	Netherlands	ATHENA national observational cohort study.	PCP occurred less frequently in patients originating from sub- Saharan Africa compared to patients of Western origin (Western Europe, New Zealand, Australia); adjusted Odds Ratio = 0.21 (0.15–0.29).
*Southern Europe*				
Varela, *et al* [38]	2004	Seville, Spain	Single centre cohort study. HIV-positive and HIV-uninfected patients with PCP.	Inverse correlation between incidence of PCP and mean ambient temperature. Highest number of cases in winter (January-March, with peak in February) when mean minimum temperature was 7–9.7**°**C (and average temperature was 10–16.5 **°**C*). No association with humidity.
Calderon, *et al* [39]	2004	Anadalusia, Spain	Thirty two public hospitals in southern Spain. HIV-positive and HIV-uninfected patients with PCP.	Seasonal variation in PCP: highest in winter (December - February: 30.5% of all cases), compared with spring (23.9%), summer (23.7%), and autumn (21.9%). Additional peak in May.
Alvaro-Meca, *et al* [39]	2015	Spain	Retrospective national study of hospitalized HIV-positive patients with PCP.	Most cases of PCP occurred in winter (December - February), fewest in summer (June - August), corresponding to lowest (10– 20 **°**C) and highest temperatures, respectively.
*Australia*				
Tadros, *et al* [40]	2017	Melbourne, Australia	Retrospective single center study of patients with systemic autoimmune rheumatic disease.	PCP most commonly presented in Autumn (March - May) when average temperature was 13.2–20°C; no cases occurred in Winter (June - August) when average temperature was 10– 12°C*.

Key: MACS = Multi-center AIDS cohort study; MSM = men who have sex with men; PCP = *Pneumocystis jirovecii* pneumonia; CDC = Centers for Disease Control and Prevention; ATHENA = AIDS Therapy Evaluation in the Netherlands; Msg = major surface glycoprotein; PCR = polymerase chain reaction; mt LSU rRNA = mitochondrial large subunit ribosomal RNA.

* data from World Weather Online [7].

**Table 3:** Human studies showing associations between *Pneumocystis* and ambient air pollution factors.

Study authors	Year	Location	Methods	Main findings
Djawe, *et al* [6]	2013	San Francisco, USA	Single center prospective cohort study of hospitalised HIV-infected patients with PCP. Climatologic and air pollution data.	Increases in SO_2_ levels associated with presentation with PCP. Effect of SO_2_ attenuated by rises in CO levels.
Blount, *et al* [42]	2013	San Francisco, USA	Single center prospective cohort study of hospitalised HIV-infected patients with PCP. Climatologic and air pollution data.	Increasing exposure to PM_10_ and NO_2_ independently associated with reduced serum IgM responses to *P. jirovecii* Msg.
Alvaro-Meca, *et al* [40]	2015	Spain	National study of hospitalized HIV-positive patients with PCP. Climatologic and air pollution data.	Higher concentrations of NO_2_, PM_10_, CO, and ozone associated with increased likelihood of hospitalization with PCP.
Blount, *et al* [43]	2017	San Francisco, USA	Single center prospective cohort study. Climatologic and air pollution data.	Increasing exposure to ozone associated with reduced BAL fluid IgA responses to *P. jirovecii* Msg, and NO_2_ independently associated with increased BAL fluid IgA responses to *P. jirovecii* Msg.

Key: HIV = human immunodeficiency virus; PCP = *Pneumocystis* pneumonia; SO_2_ = sufur dioxide; CO = carbon monoxide; NO_2_ = nitrogen dioxide; PM_10_ = particulate matter <10 μm in diameter; Msg = major surface glycoprotein; BAL = Bronchoalveolar lavage.
